# Pediatric palliative care

**DOI:** 10.1186/1824-7288-34-4

**Published:** 2008-12-01

**Authors:** Franca Benini, Marco Spizzichino, Manuela Trapanotto, Anna Ferrante

**Affiliations:** 1 Department of Pediatrics, University of Padua, Padua, Italy; 2 Ministry of Public Health, Rome, Italy

## Abstract

The WHO defines pediatric palliative care as the active total care of the child's body, mind and spirit, which also involves giving support to the family. Its purpose is to improve the quality of life of young patients and their families, and in the vast majority of cases the home is the best place to provide such care, but for cultural, affective, educational and organizational reasons, pediatric patients rarely benefit from such an approach. In daily practice, it is clear that pediatric patients experience all the clinical, psychological, ethical and spiritual problems that severe, irreversible disease and death entail. The international literature indicates a prevalence of incurable disease annually affecting 10/10,000 young people from 0 to 19 years old, with an annual mortality rate of 1/10,000 young people from birth to 17 years old. The needs of this category of patients, recorded in investigations conducted in various parts of the world, reveal much the same picture despite geographical, cultural, organizational and social differences, particularly as concerns their wish to be treated at home and the demand for better communications between the professionals involved in their care and a greater availability of support services. Different patient care models have been tested in Italy and abroad, two of institutional type (with children staying in hospitals for treating acute disease or in pediatric hospices) and two based at home (the so-called *home-based hospitalization *and *integrated home-based care *programs). Professional expertise, training, research and organization provide the essential foundations for coping with a situation that is all too often underestimated and neglected.

## Introduction and definition

The incidence of incurable disease and disability has been increasing in the Western world in recent years. Medical and technological advances have certainly reduced neonatal and pediatric mortality rates, but they have also led to a longer survival of patients with severe and potentially lethal diseases, without always succeeding in curing them, thereby increasing in absolute terms the number of pediatric patients with incurable diseases who continue to suffer from life-threatening problems.

Children with life-limiting and life-threatening illnesses that lead to death or a life of severe disability deserve a profound cultural and organizational reappraisal of how we care for them when the aim of care is not to make them recover, but to offer the best possible "health" and "quality of life", despite their disease.

Infants, children and adolescents with life-limiting and life threatening illnesses need support and long-term care in a palliative setting.

The World Health Organization defines palliative care for children as "the active total care of the child's body, mind and spirit, ... also involves giving support to the family. It begins when illness is diagnosed, and continues regardless of whether or not a child receives treatment directed at the disease" [1]. Pediatric palliative care is concerned with the medical, psycho-social, spiritual and economic needs of patients and their families, providing complex patient care solutions involving all aspects of the health care system, from hospital to hospice, to community, to home, and it involves an interdisciplinary team of caregivers.

It is important to draw a distinction between palliative care and terminal care: the latter refers to looking after children and their parents during a time closely related to their death (within weeks, days or hours). Terminal care is not palliative care, but palliative care includes terminal care [1].

The gross misconception relating to this difference heavily influences the errors made in establishing the related eligibility criteria, the needs and the best way to provide adequate solutions, especially in the pediatric setting.

For a long time, pediatric patients were not offered palliative care and, even nowadays, only a minimal part of the children with incurable disease can benefit from palliative care in Europe [2].

Numerous studies have confirmed that symptom management at the end of a pediatric patient's life is often still largely inadequate and any treatment provided is scarcely effective [3-5]. Like the clinical problems, the psychological, social and spiritual problems also receive little attention. The global situation is one of undertreatment and a scarce availability of proper patient care.

Several studies have provided important information on the multiple needs of children with life-limiting and life-threatening illnesses and their families, and on the problem of finding adequate solutions [2]. These studies have all revealed a surprisingly similar picture, despite geographical, cultural, organizational and social differences [1], i.e.

- families want their child to be treated at home and to be able to stay there until they die;

- the child wants to stay at home;

- insufficient resources currently dedicated to pediatric palliative care;

- the availability of "support" services is essential and currently inadequate;

- access to pediatric palliative care services often depends on where a child lives and the type of disease involved (they are more readily available for cancer patients);

- communication between the various professionals taking care of children with incurable disease is limited and needs to be improved;

- there is an urgent need to provide training for professionals and volunteers involved in caring for the children and their families.

Various cultural, affective, educational and organizational reasons have probably given rise to and influenced the persistence of this shortcoming in patient care. Moreover, it is by no means easy to deal with this complex problem, which demands an interdisciplinary expertise to come up with effective, realistic, applicable solutions. As is often the case, the very complexity of the problem delays the search for adequate solutions and generates doubts and controversies as to the real needs.

## Problems and peculiarities

Children make very special patients: this is already true when it comes to deciding a course of treatment, but even more so when we move into the palliative care setting. There are numerous issues to consider:

- the *limited numbers*: the number of pediatric cases of chronic and/or terminal disease requiring palliative care is fortunately limited, but the patients involved are scattered over a wide geographical area and this is bound to pose problems of an organizational nature, influencing the levels of available expertise, training and costs [1];

- the *variable types of disease and their duration*: the spectrum of conditions requiring pediatric palliative care is broad and heterogeneous (including neurological, oncological, metabolic, chromosomal, cardiological, respiratory and infectious diseases, the effects of prematurity and trauma, etc.), and the duration and complexity of the patient care required are equally varied [1]. Many pediatric diseases are also rare, and a fair proportion of cases remain without a diagnosis;

- the *specificity and complexity of the measures required*: despite their limited numerosity, these patients need very different types of approach, for various periods of time and entail an emotional involvement that amplify the energy demands and call for a multidisciplinary action of a highly complex nature. Children are in continuous physical, emotional and cognitive evolution, and this affects every aspects of their care, from the dosage of medication to the choice of methods for communication, containment and support;

- the *novelty of the problem*: in some cases, the need to extend palliative care to the pediatric setting is a consequence of technological advances enabling the survival (even in the longer term) of patients with complex diseases that led relatively rapidly to the patient's death up until not many years ago. The novelty of the problem gives rise to cultural shortcomings and training inadequacies and helps to explain why it is so difficult to provide the specific expertise needed by the healthcare and other operators;

- the *role of family*: all children are members of a close-knit unit consisting of their family, which has a very special role when young patients develop incurable, chronic disease. Parents legally represent their offspring in all clinical, therapeutic, ethical and social decisions. The family has a pivotal role in communications with healthcare providers and institutions, and if the child remains at home, then patient care and treatment is delegated to the family. Other members of the expanded family also play an important part in creating a sharing and affective support network with a cascade effect on the sick children's and their parents' quality of life [1];

- the *emotional involvement*: the emotional and affective involvement accompanying the course of disease when it is a child who is dying is inescapable, making it more difficult for family members and healthcare operators to accept the failure of treatment, the irreversibility of the disease and death, and it often becomes easier to slip into situations of overtreatment or the refusal of treatment;

- the *ethical and legal issues*: children have a very particular ethical and social position in our society. When the patient is a child, it is not always easy to speak of freedom of choice, respect for the patients' wishes, and their right to honest communications. The legal reference is the child's parent or guardian. In the pediatric setting, more than in any other, there may be a marked dichotomy between the dictates of professional ethics and deontology, and those of legislation.

All these issues combine to influence the type and entity of very particular needs, that can only be met by implementing highly specific organizational decisions and patient care models.

## Eligibility criteria

According to the literature, life-limiting and life-threatening diseases are eligible for pediatric palliative care [1].

The term *life-limiting *defines a condition where premature death is usual (e.g. Duchenne muscular dystrophy, trisomy 18, trisomy 22), while a *life-threatening *condition carries a high likelihood of premature death due to severe illness, but there is also a chance of long-term survival into adulthood (e.g. cancer).

Four different categories of childhood diseases have been identified by the Association for Children with Life-threatening or Terminal Conditions and the Royal College of Paediatrics and Child Health [6]:

1. life-threatening conditions for which curative treatment may be feasible but can fail, where palliative care is provided together with attempts at curative treatment. Examples: cancer, irreversible organ failure;

2. diseases which are life-threatening at an early age, where appropriate treatment may prolong life and provide an adequate quality of life. Examples: cystic fibrosis;

3. progressive conditions without curative treatment options, where treatment is exclusively palliative. Examples: some chromosomal diseases, muscular dystrophy, rare metabolic diseases;

4. non-progressive, irreversible conditions, with complex healthcare needs, that give rise to many complications and premature death. Examples: severe cerebral palsy, brain or spinal cord injuries due to trauma or infection.

Clearly, therefore, how long palliative care may be needed in children with incurable disease is an extremely variable parameter and hard to predict: in some cases, it is limited to the early years of life (congenital diseases); in others, it may be needed for far longer (neurological, cardiological or autoimmune diseases), and in other cases again, it may be concentrated in a brief period before death. In all these cases, however, no clear distinction is made between curative attempts to improve the patient's quality of life and extend its duration, and purely "palliative" measures; both approaches coexist and one prevails over the other depending on the stage of disease and the situation.

For the time being, the absence of a curative therapy and the presence of complex clinical, psycho-relational, social and spiritual needs demanding a multi-specialistic approach are the elements that define eligibility and drive the implementation of specialist palliative care.

## Epidemiological data

There are currently no published data on the incidence and prevalence of life-limiting and life-threatening pediatric disease in the majority of European countries, but it is essential to obtain information on the numbers, diagnostic categories, age ranges and location of children with life-limiting or life-threatening conditions in order to design and organize adequate patient care solutions.

Some degree of variability is evident in analyses conducted to date on the mortality rates for life-limiting and terminal pediatric illness.

A study by the Association for Children with Life-Threatening or Terminal Conditions and their Families, and by the Royal College of Paediatrics and Child Health in England [6] identified an annual mortality due to incurable disease of 1/10,000 children aged from one to 17 years. An Italian study [7] identified a mortality rate of 1100–1200 pediatric cases a year, corresponding to 1/10,000 0–17 year-olds. Only 40% of these patients die at home, the percentage being marginally higher for cancer victims (41%) than for other types of disease (38%). In Italy, the proportion of patients dying at home varies considerably by geographical region, the figure being 60–70% in the south and 10–15% in the north of the country. A study on mortality in children with life-threatening or terminal disease in the Republic of Ireland identified an annual death rate of 3.6/10,000 patients aged from 0 to 19 years [8].

Different mortality rates are reported in infancy, childhood and adolescence. In all studies, many childhood deaths occur in the first year of life, the majority of them caused by congenital anomalies, deformities and chromosomal anomalies. Deaths occurring after the first year of life are more likely to be caused by diseases of the nervous or circulatory systems, or neoplasms [6-8].

Available reports on the prevalence of life-limiting and terminal illness in pediatric age also paint a rather heterogeneous picture.

The analysis conducted by the Association for Children with Life-Threatening or Terminal Conditions and their Families and by the Royal College of Paediatrics and Child Health in England [6] identified a prevalence of incurable diseases among 0–17 year-olds amounting to 10/10,000/year. Forty per cent of these are cases of cancer, the other 60% comprising a miscellany of conditions, particularly neurodegenerative, metabolic and genetic diseases.

An Italian study currently in press analyzed the hospital discharge reports and mortality data on pediatric patients for the years 2002–2004, recording 12,000 children with life-limiting and terminal illness, corresponding to an annual prevalence of 10/10,000 children (0–17 year-olds).

The estimated prevalence in the Irish Republic of children and young people likely to require palliative care is 16/10,000 population aged 0–19 (15/10,000 if neonatal deaths are excluded) [8].

The differences emerging in the data collected are due to differences in the age ranges considered and in the eligibility criteria adopted.

## Needs

Children with life-limiting and life-threatening disease and their families have diverse and mutable priorities that include clinical needs (symptom assessment and management, patient care planning and sharing clinical, organizational and social decisions) [4,9], psychosocial needs (of the children and their families) [1], social needs (education, play, economic support and services), and spiritual needs (of the children and their families) [1]. To provide adequate patient care, however, it is also important to contextualize and assess the needs of the team taking care of these patients and their families, and of the institutions that have to meet these new needs and find new ways to provide patient care.

### The child's needs

The priority is symptom control. The literature [3,5] confirms major shortcomings in the provision and efficacy of treatment for patients with terminal disease. Even now, most children with life-threatening and terminal illness have many, often severe symptoms that have a dramatic, negative impact on their quality of life: about 90% of them experience generalized suffering; more than 70% suffer pain, which is controlled in less than 30% of cases. Little is known about how dyspnea and respiratory distress are controlled in these patients, but the proportion of cases given effective treatment is limited to around 20%. There are numerous options available, but their application in daily clinical practice is wholly inadequate.

Needs relating to the psychological, communication, social exchange and spiritual spheres are also often not satisfied: these problems are delegated to the family, and only rarely considered part of a global patient care project [9].

In every single case, the patient's needs are continuously changing, in both intensity and prevalence, in relation to the child's natural psychological, physical and emotional development, and to the trend of the disease and its effects on the child's growth and developmental progress.

However short the patients' future life expectancy might be, and however severely these lives are affected or underdeveloped, the acquisition of new functions and abilities, and the child's development and maturity are core issues that must guide any action undertaken, from the more traditional healthcare measures (pharmacological treatments, supplementation or substitution of vital functions or organs, etc.) to those involving other sectors of society, to assure an adequate personal growth, provide an education, deliver cultural and creative input, and support the patients' spirituality and their social role in the community.

### The family's needs

The family is a fundamental part of any pediatric palliative care program: it is actively involved in providing care and has a great deal of responsibility for caring for the child, having to make often difficult decisions in the child's best interest, and paying the social and economic price of incurable disease; without adequate support, its burden is often too heavy to bear [1,10]. The needs of the family are numerous and include:

- educational needs, i.e. training on various aspects of patient care and support;

- psychological needs: they require assessment, support and treatment for sentiments such as guilt, rage, depression, anticipatory grief, and escape;

- spiritual needs: a competent source of answers, open to an exchange of ideas and respectful of people's cultural background and religious beliefs;

- financial and social needs: practical proposals for coping with a situation of isolation, loss of identity and financial insecurity for families whose members often lose their jobs, as well as supporting the cost of patient treatment and care.

The grief associated with the incurable disease and death of a child has devastating, long-term implications for the whole family. The child's siblings have special needs during the child's illness and after their death. These brothers and sisters are at high risk of subsequent problems at school and in their relationship with their parents, as well as other psychological and social problems after their sibling's death. These problems are less evident if the disease and death are managed at home rather than in hospital [11].

Other members of the expanded family (grandparents, uncles and aunts, and friends) can play an important part in creating a supportive, sharing and affective network during the child's disease and after their death: these people also need support and supervision.

### The needs of the team

- Education and training for healthcare professionals: several studies conducted in Europe and the United States have emphasized an important shortage of know-how among public health operators as regards the fundamentals of palliative care [12,13]. Various types of expertise are required: in addition to the technical abilities needed for diagnosis and treatment, it is indispensable to have skills relating to communication (with the children and their families), teamwork and service organization.

- Supervision: the emotional impact and stress are undeniable and are often considered responsible for situations of severe burnout, leading to a rapid turnover of the professionals involved at the expense of the acquisition of the necessary experience and professional skills. These people needs support, sharing and supervision to help them cope with the problems of death and incurable disease, so that they can be useful to others, without losing their own identity and personality.

- Resources: little interest has been paid so far to pediatric palliative care as a need that healthcare organizations must meet, and the professional expertise of people working in this field has received scarce acknowledgement. It is now clear to all, from the literature and in daily clinical practice, that pediatric palliative care is a fundamental issue and warrants the allocation of specific resources, and that it is currently falling very short of meeting the real needs.

- Public education: people need to learn that palliative care should be seen as serving a right to quality of life: well-informed users know what to ask for, and this facilitates the work of healthcare operators and enables better forms of cooperation and a sharing of the problems and potential solutions.

### The needs of institutions

Institutions are faced with an entirely new and complex demand for care, as regards both the type of patient and how their needs can be met, in a context where little information is available, research has been limited and much remains to be done in terms of social and healthcare programs, the availability of monitoring tools, and cost analyses [1].

It is consequently important to produce:

- epidemiological data on the numbers, diagnostic categories, age ranges and locations of children with life-limiting or life-threatening conditions, in the light of current patient care methods and the related costs;

- tools and indicators/standards for monitoring the quality of care and the quality of life of such young patients and their families;

- assessments of the best practices in pediatric palliative care in all settings and situations in which is it needed.

## Healthcare models

Responding to these needs is by no means easy and, throughout the course of the disease, from diagnosis to death and beyond, it requires a multi-specialistic, shared action on the part of various services and institutions, that can come together to act as a single reference.

Home management is the goal of pediatric palliative care, much appreciated by patients and their families, influencing the patient's quality of life, reducing the feelings of fear, isolation and helplessness, giving the child a chance to stay involved in the family's routines, and affording more opportunities for communication [1,14,15]. Home management is not always feasible, however, and particularly complex clinical problems, exhaustion, emotional stress, logistic and organizational factors sometimes make temporary periods in institutional settings unavoidable.

From an organizational standpoint, there are theoretically four possible solutions for children with life-limiting or life-threatening disease, two domestic and two institutional:

- *institutionalized pediatric palliative care*, provided in structures specifically for incurable children (hospices or nursing homes dedicated to patients with specific diseases) or in hospital departments dealing with acute conditions;

- *pediatric palliative care at home*, where patients are cared for in their own homes by a team from the hospital, or from the territorial health care services, or a combination of the two (*integrated home care *schemes).

None of these solutions is ideal; in practice, they all have their pros and cons.

The institutional solution, in a hospice or dedicated nursing home, has the advantage of focusing expertise in the management of rare and complex cases, assuring large enough numbers to enable the necessary professional competence and economically sustainable dedicated resources to be provided, but it has the disadvantage of separating the children from their environments. This approach to providing care is in contrast with the patients' and their families desire to return home and it cannot be the only solution, especially in the case of a very lengthy course of disease.

The same problems apply to managing palliative care in hospitals dealing with acute conditions; in addition, numerous reports in the literature have confirmed that hospitals for dealing with acute conditions is far removed, in terms of its mission and aptitude, its organization and the opportunities it offers, from the ideal setting for providing palliative care for children.

While hospitalization at home can certainly assure the necessary competence and continuity in patient management, it has its drawbacks relating to the fact that the operators' expertise is disease-specific and it can only afford a limited territorial coverage. Since the number of users involved is always limited, it is difficult – in organizational and economic terms – to implement these solutions outside large towns where the density of the population is greatest.

Keeping patients at home under an integrated home care system restores children to their families and social settings, enabling ample territorial areas to be covered and multidisciplinary services to be offered, but this approach often suffers from shortcomings in the continuity of the treatment and healthcare program involving the hospitals and limited resources and experience, and the burden of patient management and support frequently lies mainly with the family.

Because none of the organizational options considered is without its drawbacks, almost all pediatric palliative care models currently adopted use a combination of the four types described above, considering them almost as organizational modules within a single support network, in which various public health and other institutions are involved at different times in the course of the patient's disease, giving priority to one or other type of solution depending on a patient's conditions and specific situations.

The various experiences gained in different countries and in some parts of Italy show that the organization of a dedicated pediatric palliative care network combining home care with institutional solutions (hospices) stands as a reference in terms of its efficacy and efficiency, and its feasibility.

Since the pediatric patients requiring palliative care are fortunately few and far between, and pose complex management issues, the trials currently underway would suggest the need to create specific care networks on fairly large scale (regional or even supra-regional) with the support of a dedicated team of pediatric palliative care specialists who manage the numerous and varied needs of children with incurable disease and their families in close cooperation with other territorial and hospital care providers.

An indispensable goal of this network is to develop a capable, multidisciplinary pediatric palliative care team (including physicians, nurses, psychologists, physiotherapists, occupational therapists, and social support workers) who will serve as a unifying reference throughout the course of the disease and after the child's death, assuring availability and help around the clock, and the opportunity to access appropriate respite and immediate hospice care if necessary [1-9].

In the UK, evidence in the literature shows that using the community services to care for children is far more cost-effective than having children spend inappropriate amounts of time in hospitals or attending hospital outpatient clinics [16].

Home is the best place to manage terminal disease and the pediatric patient's social role makes this easier to do for them than for an adult. Families want their children to stay at home, partly to preserve an impression of normality, partly because that is what their child want, but they also need to feel safe and secure, they want to be in control but they also need support [17].

The pediatric hospice is an important link in the pediatric palliative care chain: it is a highly complex structure designed with the child in mind, open-space, with areas suited to specific age groups, giving the children a chance to socialize, and providing the expertise and social interactions that can help the patient lead a "normal life". It can be the starting point that leads families to manage their "special" child at home, or it can temporarily give the family a chance to take a rest from the burden of care, when the family needs help for some reason, or when the patient's clinical management becomes too complex. It is not a place where patients go just before they die; it is a reference continually interacting in the care network on clinical, training and research levels. Although a child's care may focus more and more on palliation as the disease progresses, active treatment is still part of palliative care in the hospice and both aspects play an important part throughout a child's illness.

With this type of model, the number of beds needed in pediatric hospices is distinctly limited (4–5 beds per 3 million population) [1,9,18,19].

## Practical experience

Various solutions are currently being tested. On the international scene, it is worth mentioning the experience of the network of integrated hospices linked to a satellite home care program currently underway in the United States, in a vast territorial setting that includes a number of states, and covers a large population. This network of centers is characterized by functional links, shared protocols and behavior patterns, and the pooling of information.

An Australian experience likewise focuses on the fundamental role of home care. Despite the country's vast dimensions, there are reference centers specializing in pediatric palliative care, generally based at a few pediatric hospices and working in close contact with family pediatricians, hospitals and territorial services, working as essential partners to enable very complex patients demanding high levels of care to be managed at home. Direct telecommunications between the home and the center facilitate this process and enable the child to stay at home. The computer-based infrastructure also helps them to continue with their schooling and contributes to reducing the isolation and solitude of these children and their families.

In Italy, a few regional experiences are demonstrating the feasibility and validity of patient care models based on a network of services that include specialist care and intermediate care levels.

In the Veneto region, there is a pediatric palliative care network with a pediatric hospice in Padua serving as its specialist reference center, where a multiprofessional team of palliative care specialists provide supervision, training and care integrated with the territorial and hospital services for children with cancer and other diseases in need of palliative care throughout the region (with a population of 7.5 million). In the Veneto, a diagnosis of incurable disease and complex needs in a pediatric patient (whatever the type of disease or the patient's age and place of residence) coincides with the activation of this regional centre for pediatric palliative care. This can be done by the family pediatrician or the hospital physician dealing with the case. The specialist palliative care team at the regional centre (physician, nurse, psychologist) meets the child and his family, and all the public health and other figures involved in the case. At a first meeting, the family pediatrician, the territorial services of the local public health office, the physician specializing in the patient's disease, the reference person at the pediatrics department of the hospital serving the area where the child lives and representatives from the local authority and from the specialist palliative care team assess the case, the resources available and the feasibility of meeting the clinical and other needs of the child and his family. An integrated patient care program is prepared and shared with the child (depending on his age and disease) and his parents. The home care program involves the territorial health care personnel undergoing a period of training and sharing of procedures and methods for dealing with acute problems, predictable or otherwise, which is provided at the reference centre or at the specialist department that diagnosed the child's disease. Once the child is at home, the person responsible for the case is the family pediatrician who takes care of the patient with the cooperation of the territorial services and the regional centre for specialist palliative care, which coordinates the services required, and assures technical supervision and support for all members of the expanded team to guarantee a round-the-clock medical/nursing service. Meetings are also held between the specialist palliative care team, the family pediatrician, the territorial services and all the other institutions directly and/or indirectly involved in managing these young patients and their families, to assess the efficacy of the service being provided, any changes needed, organizational problems to solve, and whether the needs of the team have been met. If a child goes into hospital or the hospice, the reference team continues its work within the residential institution too.

Albeit using different organizational methods, there are numerous other pediatric palliative care experiences ongoing in Italy and around the world: the diversity of care solutions provided (due mainly to local issues and the different availability of resources in the areas involved) is a factor that should stimulate comparisons, research and experimentation with different practical approaches to ensure that these very "special" patients are guaranteed the competent, unequivocal response and global care they need.

## Currently legislation in Italy

In legal terms, the last two years have seen considerable advances and many issues have been brought up for debate.

- In the decree of the President of the Republic of 7 April 2006 on the adoption of the Italian national public health plan for *2006–2008 *(published in the Official Gazette n.139 of 17 June 2006), the strategic objective 3.10 states that, *" particular attention must be paid to the need for palliative care in neonatal, pediatric and adolescent age, bearing in mind the considerable diversity of the problems involved by comparison with those of adult age and the elderly, the great variety and fragmentation of the diseases in question, which are often rare and demand highly-specialized measures, and the often very lengthy and unpredictable time interval during which such care is needed. Given the above considerations, it is indispensable to organize dedicated palliative care networks for this age group that can guarantee the quality and specialization of the action needed together with a global and multi-dimensional care for the children and their families."*

- The *Technical document on palliative care for the newborn, children and adolescents*, issued by the Italian Ministry of Public Health in December 2006, defines the context and the particular features, the care models currently adopted in Italy and internationally, and the necessary resources.

- The document on *Institutional and semi-institutional care provision*, approved by the Essential Assistance Levels Commissionon 13 May 2007, includes a specific section on the pediatric setting.

- The *Agreement between the State and the Regions*, approved on 27 June 2007 at the conference on palliative care in neonatal, pediatric and adolescent age, lays the foundations for the nationwide implementation of action and programs designed to assure young people with incurable disease and their families the same availability of pediatric palliative care that, whatever the patient's age and type of disease, offers practical solutions, multi-specialistic expertise, continuity of treatments and goals, support and sharing of the burden.

- *Protocol of Intent between the Italian Ministry of Public Health and the Maruzza Lefebvre d'Ovidio Foundation*, approved on 26 September 2007, for the implementation of the "Progetto Bambino" for a National Network of pediatric palliative care services.

- The *Technical document on the State-Regions agreement*, approved on 20/3/2008, defines the public health and social support settings for providing practical support in the process for implementing pediatric palliative care in all Italian regions.

This is an area of public health programming and implementation that is undeniably difficult, where there is still much to be done.

For the children involved, palliative care also represents an indispensable goal so that pediatric medicine does not stop at establishing that a treatment has failed or that a condition is incurable, but must go on to recommend the best way for the child to live with disease.
